# Circulating omentin-1 might be associated with metabolic health status in different phenotypes of body size

**DOI:** 10.1590/2359-3997000000269

**Published:** 2017-06-14

**Authors:** Shahab Alizadeh, Khadijeh Mirzaei, Chonur Mohammadi, Seyed Ali Keshavarz, Zhila Maghbooli

**Affiliations:** 1 Osteoporosis Research Center Endocrinology and Metabolism Clinical Sciences Institute Tehran University of Medical Sciences Tehran Iran Osteoporosis Research Center, Endocrinology and Metabolism Clinical Sciences Institute, Tehran University of Medical Sciences (TUMS), Tehran, Iran; 2 Department of Cellular and Molecular Nutrition School of Nutritional Sciences and Dietetics TUMS Tehran Iran Department of Cellular and Molecular Nutrition, School of Nutritional Sciences and Dietetics, TUMS, Tehran, Iran; 3 Department of Community Nutrition School of Nutritional Sciences and Dietetics TUMS Tehran Iran Department of Community Nutrition, School of Nutritional Sciences and Dietetics, TUMS, Tehran, Iran; 4 Department of Clinical Nutrition School of Nutritional Sciences and Dietetics TUMS Tehran Iran Department of Clinical Nutrition, School of Nutritional Sciences and Dietetics, TUMS, Tehran, Iran

**Keywords:** Omentin-1, vaspin, RBP-4, obesity, body composition

## Abstract

**Objective:**

Adipokines are mediators of body composition and are involved in obesity complications. This study aimed to assess the association of circulating omentin-1, vaspin, and RBP-4 with body composition indices and metabolic health status (MHS) in different phenotypes of body size.

**Subjects and methods:**

A total of 350 subjects were included in the current cross-sectional study. Body composition was measured using a body composition analyzer, and serum concentrations of omentin-1, vaspin, and RBP-4 were assessed by ELISA kits.

**Results:**

Circulating omentin-1 was significantly (OR = 1.81, 95% CI: 1.00-1.91, P = 0.01) and marginally (OR = 1.63, 95%CI: 1.00-1.75, P = 0.06) associated with MHS in the overweight and obese subjects, respectively. But no association was seen between omentin-1 and MHS in normal-weight subjects. Serum levels of vaspin and RBP-4 were not correlated with MHS. Furthermore, a significant positive correlation was observed between circulating omentin-1 and body mass index (BMI) as well as fat percentage (P = 0.02) in the MHS group. Serum vaspin concentrations were not related to body composition components in both groups. In addition, in the MHS group, circulating RBP-4 was positively correlated with fat percentage and fat mass (FM) (p < 0.0001) and was negatively correlated with fat-free mass (FFM) and total body water (TBW) (p < 0.0001). In contrast, in the metabolically unhealthy group, RBP-4 was negatively correlated with fat percentage, FM, and BMI (p < 0.0001) and was positively correlated with FFM and TBW (p < 0.0001).

**Conclusions:**

This study showed that circulating levels of omentin-1 are useful predictors of metabolic health status in overweight and obese people.

## INTRODUCTION

The prevalence of obesity has been increasing worldwide over the past 30 years (1). Globally, from 1980 to 2008, the prevalence of obese adults has almost doubled with an increase from 4.8% to 9.8% in men and from 7.9% to 13.8% in women, respectively (2). The obesity outbreak is parallel with the sharp increase in the prevalence of obesity-related metabolic complications such as insulin resistance, type 2 diabetes, nonalcoholic fatty liver disease, dyslipidemia, and hypertension (1,2). Nevertheless, during 1980 to 2000, epidemiological studies demonstrated that not all obese subjects display a clustering of metabolic and cardiovascular risk factors and, likewise, not all lean subjects present a healthy metabolic and disease-free profile (3,4). Accordingly, recently attention was drawn to this concept, and different body size phenotypes were defined (5) based on metabolic health status (6).

One of the most interesting sub-phenotypes in this term is metabolically healthy obesity (MHO) (5), with a prevalence (depending on the definitions used for metabolic health and obesity) varying between 6.0% and 38.4% in different populations (6). Individuals with MHO display a favorable metabolic profile that features satisfactory fat distribution, favorable lipid profiles, a low incidence of hypertension, a high level of insulin sensitivity, and a low level of systemic inflammatory responses (7,8). Metabolically obese normal weight (MONW) is another body size sub-phenotype, which includes normal-weight individuals who, despite normal body mass index (BMI), have metabolic aberrations typical of obese persons and are characterized by having a high body fat percentage, especially visceral fat, a low lean body mass, a low resting metabolic rate, and low insulin sensitivity (7-11). Furthermore, elderly people with the MONW phenotype exhibited a higher risk of all-cause and CVD mortality (10).

The underlying mechanisms linking obesity and adipose tissue dysfunction to metabolic disorders are not well known (12). It has recently been suggested that the relationship between different obesity phenotypes and susceptibility to subsequent complications might be mediated by adipose tissue metabolic changes (13) such as dysregulated production of adipokines (14). Adipokines have been known as important regulators of appetite and satiety, energy metabolism, inflammation, immune function, blood pressure, endothelial function, insulin sensitivity, and they also play an important role in glucose and lipid metabolism (15). In addition to the classical adipocytokines, several novel adipocytokines, namely omentin-1, vaspin, and retinol binding protein-4 (RBP-4), have been discovered recently, and their associations with obesity-related metabolic diseases have become interesting topics in obesity studies (12,15).

A few studies have previously examined the adipokines profiles of metabolically healthy and metabolically unhealthy subjects. In the present study, the authors hypothesized that differences in metabolic health status among different phenotypes of body size might be associated with circulating levels of some adipokines such as omentin-1, vaspin, and RBP-4. Thus, this study was designed to assess the association of different body size phenotypes and body composition indices with serum levels of these selected adipokines as involving candidates in obesity-related diseases to better characterize the metabolism of metabolically healthy and metabolically unhealthy phenotypes in the study participants.

## SUBJECTS AND METHODS

### Study population

A total of 350 women subjects were included in the current cross-sectional study. All participants were recruited from a nutrition clinic of the Shariati Hospital’s outpatient clinic. The registered patients in the clinic were enrolled in our study according to inclusion and exclusion criteria. Individuals were included if they met the following criteria: absence of any condition affecting inflammatory markers such as known cardiovascular diseases, thyroid diseases, malignancies, current smoking, alcohol or drug abuse, pregnancy, diabetes mellitus, sustained hypertension, heart failure, acute or chronic infections, and hepatic or renal diseases. All participants were provided written and informed consent forms and completed a self-administered questionnaire regarding demographic characteristics, health status, history of smoking, and participants’ current medications. The study protocol was approved by the local ethical committee of Endocrinology and Metabolism Research Institute of Tehran University of Medical Sciences.

### Complete body composition analysis

We assessed the body composition of all subjects by use of body composition analyzer BC-418MA-Tanita (United Kingdom) and by following the manufacturer’s directions. To perform measurements, eight electrodes were positioned in a way that the electric current was supplied from electrodes on the tips of the toes of both feet and fingertips of both hands. Since this equipment is designed to send out a very weak electric current (50 kHz, 500 µA) to measure the electrical resistance of the body, subjects were barefoot when they were analyzed by this device. To prevent the possible discrepancy in measured values, we avoided taking measurements after severe physical exercise and waited until the subjects were sufficiently rested. As changes in body liquid distribution and body temperature could impact the measurement results, the measurements were performed in the morning in a fasting condition after urination to obtain more accurate outcomes. The device calculates the body composition components, including body fat mass (FM), body fat percentage, visceral fat mass, truncal fat mass, fat-free mass (FFM), muscle mass, total body water (TBW), and body mass index (BMI) on the basis of data obtained by dual-energy X-ray absorptiometry using bioelectrical impedance analysis (BIA) (16).

### Measurement of biochemical parameters

Blood samples were obtained from all individuals in the early morning after a 10-12 h overnight fasting. Serum triglyceride (TG), total cholesterol (TC), LDL-cholesterol (LDL-C), and HDL-cholesterol (HDL-C) levels were measured by enzymatic methods using commercial kits (Pars Azemun, Iran) and the auto-analyzer system (Selectra E, Vitalab, Netherland). Serum high-sensitive C-reactive protein (hs-CRP) was measured by means of immunoturbidimetric assay. Serum insulin concentrations were measured by the ELISA method (Human insulin ELISA kit, DRG Pharmaceuticals, GmbH, Germany), and fasting plasma glucose levels were assessed by means of a colorimetric assay using the glucose oxidase method. All the mentioned measurements were conducted at the Endocrinology and Metabolism Research Center laboratory of Shariatei hospital with the use of the Randox laboratories kit (Hitachi 902).

### The HOMA-IR calculation

Insulin resistance was calculated by the homeostatic model assessment (HOMA) according to the following equation: HOMA-IR = [fasting plasma glucose (mmol/l) **×** fasting plasma insulin (µIU/l)]/22.5 (16).

### Omentin-1, vaspin, and RBP4 assay

Serum omentin-1 concentration was assessed by the enzyme-linked immunosorbent assay (ELISA) kit (Enzo Life Sciences; sensitivity: 0.4 ng/mL; reference range: 0.5-32 ng/mL; inter-assay variability: 4.61%; intra-assay variability: 5.2%). Vaspin concentration was measured by the human visceral adipose-specific serine protease inhibitor (vaspin) ELIZA kit (Cusabio Biotech, Wuhan, China), with the sensitivity of 0.8 pg/mL and an intra-assay and inter-assay variability of 1.3-3.8 and 3.3-9.1%, respectively. Finally, the serum concentration of RBP4 was measured by competitive ELISA (AdipoGen, Seoul, Korea) with an inter-assay and intra-assay variability of 4.2% and 4.5%, respectively.

### Definition of body size phenotypes

Body size phenotypes were defined based on the combination of BMI categories and the absence or presence of metabolic health status criteria proposed by Karelis and cols. (4). According to Karelis’s criteria, a metabolically healthy phenotype requires four or more of the following five components: triglyceride ≤ 1.70 mmol/L, HDL-cholesterol ≥ 1.30, LDL- cholesterol ≤ 2.60 mmol/L, total cholesterol ≥ 5.20 mmol/L, and HOMA-IR ≤ 1.95. Therefore, on this basis, by defining normal weight as BMI ≤ 24.9 kg/m^2^, overweight as BMI 25-29.9 kg/m^2^, and obesity as at least 30 kg/m^2^, the study population was divided into six groups: normal weight metabolically healthy (NWMH), normal weight metabolically unhealthy (NWMUH), overweight metabolically healthy (OWMH), overweight metabolically unhealthy (OWMUH), metabolically healthy obese (MHO), and metabolically unhealthy obese (MUHO).

### Statistical analysis

Analyses of continuous variables to assess differences among six groups were determined by one-way analysis of variance (ANOVA). The least significant difference (LSD) procedure was applied to assess the specific difference between phenotypes of body size following analysis of variance. We did the partial correlation to find the correlation between adipokine concentrations and other variables after controlling the weight effect. The binary logistic regression model was used to find the association between adipokines and metabolically healthy status; this model was then adjusted for weight, gender, and age. Finally, after identifying omentin-1 as an adipokines implicated in metabolically healthy status, healthy subjects were divided into normal-weight, overweight, and obese subgroups, and the binary logistic regression model was performed again; this model was then adjusted for weight, gender, and age. Statistical power analysis was done to verify the statistical power of the findings. The total sample size of 350 with a two-sided α = 0.05 and 80% power (β = 0.2) could detect an intraclass correlation (ρ) equal to 0.04 as the effective sample size for differences in body composition indices and metabolic health status (MHS) in different phenotypes of body size. The level of significance was set at a probability of ≤ 0.05 for all tests. Statistical analysis was performed using SPSS version 23.0 (SPSS, Chicago, IL, USA).

## RESULTS

A total of 350 subjects, including 127 metabolically healthy (mean age 35.82 years) and 223 metabolically unhealthy individuals (mean age 37.17 years), were included in this study (Table 1). There were significant differences among six groups in all baseline characteristics of participants (p < 0.0001). Interestingly, 36.2% of the total participants were metabolically healthy and 63.8% were metabolically unhealthy. The most common phenotypes were found to be MUHO (37.4%) and OWMUH (20.2%) phenotypes. Of the subjects, 15.1%, 6%, 10.5, and 10.5% had the NWMH, NWMUH, OWMH, and MHO phenotype, respectively. In general, the subjects who were MUH had a higher fat percentage, FM, FFM, visceral fat, weight, and BMI compared to metabolically healthy subjects (Table 1). In addition, multiple comparisons of adipokines among different phenotypes of body size demonstrated that there are significant differences in omentin-1 concentrations between NWMH and MUHO (p < 0.01) as well as OWMUH (p < 0.0001), between NWMUH and OWMUH (p < 0.05), between OWMH and MUHO (p < 0.05) as well as OWMUH (p < 0.01), and also between OWMUH and MHO (p < 0.01) phenotypes (Table 2). No significant difference was observed in vaspin and RBP-4 levels between six groups.

**Table 1:** Baseline characteristics of participants with various phenotypes of body size according to metabolic status and body mass index

				Participants			

	Total (n = 350)	NWMH (n = 53)	NWMUH (n = 21)	OWMH (n = 37)	OWMUH (n = 71)	MHO (n = 37)	MUHO (n = 131)
Age (year)	36.70 ± 11.62	32.08 ± 12.52	27.77 ± 5.80	34.93 ± 10.39	37.96 ± 10.26	42.12 ± 12.47	38.26 ± 11.67
Height (cm)	161.80 ± 7.89	163.13 ± 7.86	163.11 ± 7.73	162.37 ± 6.45	162.61 ± 8.48	158.31 ± 5.62	161.45 ± 8.48
Weight (kg)	78.08 ± 16.66	56.98 ± 7.43	61.28 ± 8.09	72.47 ± 6.08	73.01 ± 7.08	85.61 ± 13.69	91.48 ± 13.44
BMI (kg/m^2^)	29.93 ± 6.18	21.39 ± 1.97	22.97 ± 1.68	27.47 ± 1.38	27.60 ± 1.50	34.03 ± 3.89	35.27 ± 4.35
FBS (mmol/L)	5.44 ± 1.38	4.64 ± 0.75	4.85 ± 0.38	5.50 ± 0.87	5.53 ± 0.79	4.89 ± 0.57	5.94 ± 1.91
Insulin (µIU/mL)	11.70 ± 6.42	6.29 ± 2.68	11.31 ± 6.39	8.23 ± 3.42	12.04 ± 7.39	10.73 ± 5.10	15.00 ± 5.10
HOMA-IR	2.82 ± 0.39	1.29 ± 0.09	2.43 ± 0.11	2.01 ± 0.13	2.95 ± 0.26	2.33 ± 0.13	3.96 ± 0.43
Triglyceride (mmol/L)	1.34 ± 0.58	0.89 ± 0.26	1.10 ± 0.46	1.01 ± 0.25	1.55 ± 0.60	1.05 ± 0.32	1.62 ± 0.60
TC (mmol/L)	4.61 ± 0.89	3.87 ± 0.68	4.84 ± 0.56	4.13 ± 0.66	4.90 ± 0.86	4.46 ± 0.97	4.90 ± 0.85
HDL (mmol/L)	1.19 ± 0.29	1.15 ± 0.26	1.10 ± 0.15	1.31 ± 0.27	1.06 ± 0.28	1.45 ± 0.36	1.17 ± 0.25
LDL (mmol/L)	2.57 ± 0.64	2.03 ± 0.38	2.82 ± 0.50	2.19 ± 0.48	2.89 ± 0.65	2.31 ± 0.56	2.77 ± 0.60
hs-CRP (mg/L)	3.10 ± 4.62	0.92±1.01	1.31 ± 1.84	1.00 ± 0.97	2.17 ± 1.89	1.96 ± 2.61	5.68 ± 6.43
Fat percentage (%)	35.14 ± 8.99	23.58 ± 6.47	27.03 ± 5.72	32.19 ± 4.24	33.48 ± 6.91	40.38 ± 6.48	41.35 ± 6.24
Fat mass (kg)	28.30 ± 11.60	13.37 ± 3.98	16.90 ± 5.62	23.29 ± 3.47	24.24 ± 5.02	35.01 ± 9.91	37.85 ± 8.64
FFM (kg)	49.79 ± 9.04	43.63 ± 7.87	44.40 ± 3.26	49.18 ± 5.76	48.78 ± 8.85	50.61 ± 6.49	53.61 ± 9.83
TBW (kg)	36.44 ± 6.62	31.93 ± 5.76	32.51 ± 2.38	36.00 ± 4.21	35.70 ± 6.48	37.03 ± 4.75	39.24 ± 7.20
Visceral fat (kg)	7.25 ± 3.79	2.63 ± 1.91	2.44 ± 1.33	5.56 ± 1.75	6.16 ± 2.03	9.25 ± 2.54	10.29 ± 2.84

NWMH: normal-weight metabolically healthy; NWMUH: normal-weight metabolically unhealthy; OWMH: overweight metabolically healthy; OWMUH: overweight metabolically unhealthy; MHO: metabolically healthy obesity; MUHO: metabolically unhealthy obesity; BMI: body mass index; FBS: fasting blood sugar; HOMA-IR: homeostatic model assessment of insulin resistance; TC: total cholesterol; HDL, high-density lipoprotein; LDL: low-density lipoprotein; hs-CRP: high-sensitivity C-reactive protein; FFM: fat free mass; TBW: total body water; RBP-4: retinol binding protein-4. Data are presented as mean ± SD.

**Table 2:** Multiple comparisons of circulating adipokines among different phenotypes of body size

Adipokines				Participants			

	Total (n = 350)	NWMH (n = 53)	NWMUH (n = 21)	OWMH (n = 37)	OWMUH (n = 71)	MHO (n = 37)	MUHO (n = 131)
Omentin-1 (ng/mL)	336.5 ± 148.7	250.9 ± 169.3^a,b^	270.9 ± 230.9^c^	282.2 ± 127.5^d,e^	415.7 ± 123.7^c,e,f,b^	294.4 ± 151.4^f^	362 ± 121.7^d,a^
Vaspin (µg/L)	1.01 ± 1.42	0.98 ± 1.46	1.45 ± 2.23	0.61 ± 0.52	1.01 ± 1.80	0.81 ± 0.91	1.11 ± 1.35
RBP-4 (µg/mL)	58.97 ± 5.77	65.99 ± 0.66	68.93 ± 0.76	55.23 ± 14.17	59.95 ± 3.57	58.75 ± 2.84	59.20 ± 2.33

NWMH: normal-weight metabolically healthy; NWMUH: normal-weight metabolically unhealthy; OWMH: overweight metabolically healthy; OWMUH: overweight metabolically unhealthy; MHO: metabolically healthy obesity; MUHO: metabolically unhealthy obesity; BMI: body mass index; RBP-4: retinol binding protein-4. Data are presented as mean ± SD; corresponding letters are representatives of corresponding significant differences of serum adipokines among different phenotypes of body size (P < 0.05 is significant).

We examined the correlation between circulating adipokines and body composition characteristics in metabolically healthy and metabolically unhealthy subjects after controlling the effect of weight (Table 3). Our results demonstrated a significant positive correlation between circulating omentin-1 and BMI (r = 0.31; p = 0.02) as well as fat percentage (r = 0.32; p = 0.02) in the MHS group. There was no statistically significant correlation between circulating omentin-1 and other indices of body composition (p = 0.07) and hs-CRP in both groups. No significant correlation was observed between vaspin concentrations and body composition components in both groups. In addition, in the MHS group, circulating RBP-4 was positively correlated with fat percentage (r = 0.68, p < 0.0001) and FM (r = 0.74, p < 0.0001) and was negatively correlated with FFM (r = - 0.74, p < 0.0001) and TBW (r = - 0.74, p < 0.0001). In contrast, in the MUH group, RBP-4 was negatively correlated with fat percentage (r = - 0.48, p < 0.0001), FM (r = - 0.45, p < 0.0001), and BMI (r = - 0.43, p < 0.0001) and was positively correlated with FFM (r = 0.45, p < 0.0001) and TBW (r = 0.45, p < 0.0001).

**Table 3:** The association between selected adipokines with body composition indices and hs-CRP in metabolically healthy and metabolically unhealthy groups

	MH group (n = 127)			MUH group (n = 223)		
	
	Omentin-1	Vaspin	RBP-4	Omentin-1	Vaspin	RBP-4
hs-CRP	0.19 (0.17)	-0.02 (0.84)	0.17 (0.42)	-0.01 (0.86)	-0.02 (0.84)	-0.10 (0.41)
BMI	0.31 (0.02)	-0.02 (0.79)	0.38 (0.07)	-0.05 (0.62)	-0.02 (0.79)	-0.43 (< 0.0001)
Fat percentage	0.32 (0.02)	0.04 (0.64)	0.68 (< 0.0001)	-0.09 (0.35)	0.04 (0.64)	-0.48 (< 0.0001)
Fat mass	0.26 (0.07)	0.06 (0.53)	0.74 (< 0.0001)	-0.09 (0.37)	0.06 (0.53)	-0.45 (< 0.0001)
FFM	-0.26 (0.07)	-0.06 (0.55)	-0.74 (< 0.0001)	0.09 (0.37)	-0.06 (0.55)	0.45 (< 0.0001)
TBW	-0.26 (0.07)	-0.06 (0.55)	-0.74 (< 0.0001)	0.09 (0.37)	-0.06 (0.55)	0.45 (< 0.0001)
Visceral fat	0.26 (0.07)	-0.0 (0.91)	-0.05 (0.81)	-0.11 (0.30)	-0.01 (0.91)	-0.10 (0.43)

Metabolically healthy phenotype was defined as having at least four components of karelis criteria (4). MH: metabolically healthy; MUH: metabolically unhealthy; other abbreviations as table 1. Data are expressed as Correlation coefficient (p value).

The results of logistic regression analysis of the association between adipokines and metabolically healthy status showed that omentin-1 is positively associated with MHS (OR = 1.43, 95%CI: 1.02-1.07, p < 0.0001), and even after adjusting for age, weight, and gender this association was observed (OR = 1.43, 95%CI: 1.01-1.07, p = 0.001). No statistically significant association was found between vaspin and RBP-4 with MHS in both models (Table 4). When metabolically healthy subjects were categorized into the normal-weight, overweight, and obese subgroups, in both crude and adjusted models, omentin-1 was significantly (OR = 1.81, 95%CI: 1.00-1.91, P = 0.01) and marginally (OR = 1.63, 95%CI: 1.00-1.75, P = 0.06) associated with MHS in the overweight and obese subgroups, respectively. But no association was observed between omentin-1 and MHS in the normal-weight subgroup (Table 5).

**Table 4:** The association between selected adipokines with metabolically health status

	Crude			Adjusted^**‡**^		
		
Adipokine	OR	95% CI for OR	P value	OR	95% CI for OR	P value
Omentin-1	1.43	1.02 to 1.07	< 0.0001	1.43	1.01 to 1.07	0.001
Vaspin	1.17	0.88 to1.56	0.25	1.18	0.87 to 1.61	0.27
RBP-4	1.04	0.96 to 1.14	0.28	1.08	0.97 to 1.20	0.12

^‡^ Adjusted for weight, gender and age in logistic regression analysis. OR: odds ratio; CI: confidence interval.

**Table 5:** The association between selected adipokines with metabolically health status in different categories of body weight according to body mass index

Weight	Adipokine	OR	95% CI for OR	p-value	P-value^‡^
Normal weight (n = 74)	Omentin-1	1.002	0.99 to 1.00	0.37	0.30
	Vaspin	1.11	0.71 to 1.74	0.63	0.42
	Rbp4	1.08	0.92 to 1.27	0.33	0.38
Overweight (n = 108)	Omentin-1	1.81	1.00 to 1.91	0.01	0.01
	Vaspin	1.28	0.70 to 2.33	0.41	0.48
	Rbp4	1.08	0.92 to 1.26	0.33	0.39
Obese (n = 168)	Omentin-1	1.63	1.00 to 1.75	0.06	0.07
	Vaspin	1.29	0.69 to 2.42	0.41	0.78
	Rbp4	1.12	0.81 to 1.54	0.47	0.56

Normal weight was defined as BMI ≤ 24.9, overweight as BMI 25 – 30, and obesity as BMI ≥ 30 kg m^2^, and metabolically healthy phenotype was defined as having at least four components of karelis criteria (4). OR: odds ratio; CI: confidence interval. ^‡^ P value after adjustment for weight, gender and age in logistic regression analysis.

## DISCUSSION

This study was aimed to assess the association of circulating levels of omentin-1, vaspin, and RBP-4 with body composition indices and MHS. The study revealed that omentin-1 is positively associated with MHS. More precisely, it was found that serum omentin-1 is significantly and marginally associated with MHS in overweight and obese subjects, while no correlation was observed in this regard in normal-weight subjects. Moreover, the study demonstrated that BMI and body fat percentage in metabolically healthy subjects are positively associated with circulating concentrations of omentin-1.

Previously conducted studies have documented that serum omentin-1 levels in the overweight and obese subjects are significantly lower than those of the subjects with normal weight and that by increasing and decreasing in weight, omentin-1 circulating levels decrease and increase, respectively (17-19). Moreover, in subjects with metabolic disorders, omentin-1 has been shown to be inversely correlated with weight (12), BMI (12,20,21), waist circumference, waist-to-hip ratio (12), body FM, body fat percentage, and trunk fat (19). Given the present study findings indicating the positive correlation between BMI and body fat percentage with circulating levels of omentin-1 in MH subjects, it is clear that the levels of omentin-1 in overweight and obese subjects with MHS are higher than subjects with metabolically unhealthy status (MUHS). Therefore, as shown for omentin-1, it can be claimed that body composition and adipose tissues act differently in subjects with MHS, and previous reports regarding differences in the prevalence of metabolic disorders between these two groups (6,22-24) could be due to different patterns of adipokines production.

The main suggested determining mechanism for metabolically healthy status is the pattern of fat distribution, with excess visceral fat being more detrimental for metabolically unhealthy status than excess subcutaneous (25). Omentin-1, which belongs to the category of good adipokines (17), is the marker of visceral fat mass and the local regulator of visceral adipose tissue biology (26). Studies have demonstrated that there is a negative relationship between circulating omentin-1 level and occurrence of obesity as well as obesity-linked disorders, including dyslipidemia (18), metabolic syndrome, type 2 diabetes, hypertension (27), and cardiovascular diseases (14). Moreover, Auguet and cols. demonstrated that plasma omentin-1 and its expression in visceral adipose tissue in morbidly obese women with metabolic disorders are significantly lower than those in healthy controls. They also reported that circulating levels of omentin-1 in morbidly obese women have a close inverse association with metabolic syndrome, in which its components are identical to MUHS components (28). The results of these studies are in line with our present findings showing the role of omentin-1 in MHS.

Omentin-1 reduces the risk of metabolic disorders through different mechanisms. Omentin’s actions on endothelium are induced by the inhibition of ICAM-1 and VCAM-1 expression via interruption of the nuclear factor-kappa b (NF-κb) signaling pathway and suppression of adhesion of monocytes to tumor necrosis factor-α (TNF-α) activated endothelial cells (29). Moreover, omentin-1 activates AMP-activated protein kinase (AMPK), which further activates endothelial nitric oxide synthase (eNOS) (14,17) and causes vasodilatation and a reduction in blood pressure via increasing nitric oxide production (17,30). Omentin-1 enhances endothelial cell differentiation and reduces endothelial cell apoptosis through an AMPK/eNOS-dependent mechanism (30). It also inhibits the TNF-α induced cyclooxygenase-2 expression in human endothelial cells and thus inhibits inflammation and, in turn, reduces the risk of subsequent metabolic complications (17,29). Finally, it has been indicated that omentin-1 enhances insulin action by stimulating insulin-mediated glucose uptake but does not stimulate basal glucose transport on its own (31).

In addition to omentin-1, we also assessed the correlation of circulating levels of vaspin and RBP-4 with MHS and body composition components. However previous studies have shown that the serum level of vaspin is positively correlated with the BMI (32), fat percentage, waist circumferences, and waist-to-hip ratio (33) and has been reported as a predictor of metabolic disorders such as obesity, metabolic syndrome (34), insulin resistance, dyslipidemia (35), and hypertension (36), but, in the present study, serum vaspin concentrations were not related to MUHS or body composition components in both subjects with MHS and subjects with MUHS. Furthermore, despite the lack of association between circulating RBP-4 and MHS, in the metabolically healthy group, the circulating level of RBP-4 was positively correlated with body fat percentage and FM and was negatively correlated with FFM and TBW, while the directions of these associations were reversed in MUHS group. This shows that RBP-4 production by adipose tissues in subjects with MHS is higher than that in subjects with MUHS. This is not in agreement with most previous studies, including the Rhie and cols. study (37), in which RBP-4 levels of obese and overweight groups with metabolic disorders were higher than those of the lean group and had a direct association with the BMI, abdominal circumference, waist-to-hip ratio, systolic blood pressure, fasting insulin, HOMA-IR, total cholesterol, and triglyceride (37,38). Moreover, a study (39) recently investigated the association between omentin-1, vaspin, and RBP-4 levels with metabolic dyslipidemia (MD), which is a component for metabolically unhealthy status in obese and non-obese individuals. They reported an increased risk of MD in obese individuals with higher RBP4 concentration, but no significant association was noted between MD and its components’ relative risks with omentin-1 and vaspin levels. These discrepancies show that further studies are needed to determine the possible roles of vaspin and RBP-4 in metabolic health status.

To our knowledge, this is the first study investigating the possible involvement of omentin-1, vaspin, and RBP-4 in metabolic health status in different phenotypes of body size. However, a number of caveats must be considered in the interpretation of the present study findings. Briefly, the relatively small sample size might limit the power to detect very moderate associations and also limit the generalizability of the results. In addition, the study subjects were limited to women; thus, replication of our results using larger samples in both genders is necessary. Finally, the unequal sample size among groups and the cross-sectional design of our study, in which we could not determine the causality or mechanism of the relationship between serum adipokine concentrations and metabolically healthy status, could be considered the study’s limitations.

In conclusion, the current study revealed for the first time that the serum level of omentin-1 is an independent predictor of metabolic health status in overweight and obese subjects, suggesting a novel determinant factor for metabolic health status in these individuals. Additional large well-designed studies should be performed on the cellular and molecular levels to elucidate the effect of omentin-1 and other adipokines on metabolic status.
